# Partnering essential oils with antibiotics: proven therapies against bovine *Staphylococcus aureus* mastitis

**DOI:** 10.3389/fcimb.2023.1265027

**Published:** 2023-09-15

**Authors:** Marwa I. Abd El-Hamid, Reham M. El-Tarabili, Mosa M. Bahnass, Mohammed Abdulrahman Alshahrani, Ahmed Saif, Khairiah Mubarak Alwutayd, Fatmah Ahmed Safhi, Abdallah Tageldein Mansour, Noaf Abdullah N. Alblwi, Mohammed M. Ghoneim, Ayman Abo Elmaaty, Helal F. Al-harthi, Mahmoud M. Bendary

**Affiliations:** ^1^ Department of Microbiology, Faculty of Veterinary Medicine, Zagazig University, Zagazig, Egypt; ^2^ Department of Bacteriology, Immunology and Mycology, Faculty of Veterinary Medicine, Suez Canal University, Ismailia, Egypt; ^3^ Department of Animal Medicine (Infectious Diseases), Faculty of Veterinary Medicine, Zagazig University, Zagazig, Egypt; ^4^ Department of Clinical Laboratory Sciences, Applied Medical Sciences College, Najran University, Najran, Saudi Arabia; ^5^ Department of Clinical Laboratory Sciences, Faculty of Applied Medical Sciences, Najran University, Najran, Saudi Arabia; ^6^ Department of Clinical Laboratory Sciences, College of Applied Medical Sciences, King Khalid University, Abha, Saudi Arabia; ^7^ Department of Biology, College of Science, Princess Nourah bint Abdulrahman University, Riyadh, Saudi Arabia; ^8^ Animal and Fish Production Department, College of Agricultural and Food Sciences, King Faisal University, Al-Ahsa, Saudi Arabia; ^9^ Fish and Animal Production Department, Faculty of Agriculture, Alexandria University, Alexandria, Egypt; ^10^ Al Hadithah General Hospital, Al-Qurayyat, Saudi Arabia; ^11^ Department of Pharmacy Practice, College of Pharmacy, Al Maarefa University, Riyadh, Saudi Arabia; ^12^ Medicinal Chemistry Department, Faculty of Pharmacy, Port Said University, Port Said, Egypt; ^13^ Biology Department, Turabah University College, Taif University, Taif, Saudi Arabia; ^14^ Department of Microbiology and Immunology, Faculty of Pharmacy, Port Said University, Port Said, Egypt

**Keywords:** carvacrol, linalool, eugenol, MDR, MRSA, antibiofilm

## Abstract

**Introduction:**

There is an urgent need to develop therapeutic options for biofilm-producing Staphylococcus aureus (S. aureus). Therefore, the renewed interest in essential oils (EOs), especially carvacrol, linalool and eugenol, has attracted the attention of our research group.

**Methods:**

Multidrug resistance and multivirulence profiles in addition to biofilm production of S. aureus strains isolated from cows with mastitis were evaluated using both phenotypic and genotypic methods. The antimicrobial and antibiofilm activities of EOs were tested using both in vitro and molecular docking studies. Moreover, the interactions between commonly used antibiotics and the tested EOs were detected using the checkerboard method.

**Results:**

We found that all our isolates (n= 37) were biofilm methicillin resistant S. aureus (MRSA) producers and 40.5% were vancomycin resistant S. aureus (VRSA). Unfortunately, 73 and 43.2% of the recovered MRSA isolates showed multidrug resistant (MDR) and multivirulence patterns, respectively. The antimicrobial activities of the tested EOs matched with the phenotypic evaluation of the antibiofilm activities and molecular docking studies. Linalool showed the highest antimicrobial and antibiofilm activities, followed by carvacrol and eugenol EOs. Fortunately, synergistic interactions between the investigated EOs and methicillin or vancomycin were detected with fractional inhibitory concentration index (FICI) values ≤ 0.5. Moreover, the antimicrobial resistance patterns of 13 isolates changed to sensitive phenotypes after treatment with any of the investigated EOs. Treatment failure of bovine mastitis with resistant S. aureus can be avoided by combining the investigated EOs with available antimicrobial drugs.

**Conclusion:**

We hope that our findings can be translated into a formulation of new pharmaceutical dosage forms against biofilm-producing S. aureus pathogens.

## Introduction

*Staphylococcus aureus* (*S. aureus*) is a widely recognized bacterium that can spread to humans and animals resulting in life-threatening illnesses. It is also a major cause of bovine mastitis in cattle, buffalo, sheep and goats (Kløve et al., 2022). In dairy farms, *S. aureus* mastitis and its produced toxins cause major economic losses including decreased milk production, excessive drug residues contamination and chronic illness leading to deaths (Algammal et al., 2020). The antimicrobial resistance rates among mastitis *S. aureus* in Egypt are increased during the last few decades (El-Jakee et al., 2011; Ameen et al., 2019). Methicillin-resistant *Staphylococcus aureus* (MRSA) and vancomycin-resistant *Staphylococcus aureus* (VRSA), commonly called superbugs, are among the most significant pathogens that pose major threats to both human and animal health (Shrestha et al., 2021). Unfortunately, most β -lactam antibiotics are ineffective against *S. aureus* isolates, which harbors *mecA/mecC* genes (Hiramatsu et al., 2014; Saber et al., 2022). The fact that MRSA may produce biofilms on biotic and abiotic surfaces (Ascioferro et al., 2021) renders the issue even more challenging to be eradicated. It has been known for a considerable time that staphylococcal isolates are the most common causes of infections that are connected with biofilms (Lebeaux et al., 2013). The extracellular matrix, changing metabolic states and growth rate render biofilms more resistant to antibiotics than planktonic organisms (Silva et al., 2021). Notably, MRSA strains biofilm development and multidrug resistant (MDR) profile increase the possibility of chemotherapeutic failure (Abd-El-Hamid et al., 2020).

Moreover, virulence arrays are essential for overcoming the host defense power and increasing bacterial pathogenicity. Most *S. aureus* mastitis strains are multivirulent and are associated with biofilm production. The biofilm associated protein (BAP) is correlated with the presence of *bap* gene and *ica* operon, which control polysaccharide intercellular adhesin synthesis producing an extremely structured multicellular biofilm (Zhang et al., 2018). Moreover, *S. aureus* enterotoxins (SEs) and hemolysins (Hlα and Hlβ) promote pathogenicity by enhancing the pathogen’s adhesion, colonization and tissue invasion (Puah et al., 2016). Expression of *S. aureus* virulence genes is controlled via accessory gene regulatory (*agr*) system, which can be divided into four groups according to *agrC* and *agrD* gene sequences; *agr* I, II, III and IV. Although *agr* types vary in properties and prevalence according to geography, identifying the dominant type in each region may be beneficial (Javdan et al., 2019).

Intramammary infections caused by biofilm-producing *S. aureus* are common among cows with chronic mastitis. The incidence of these infections is increasing with poor management practices during milking. Milk is an excellent medium for the growth of bacterial species gaining access to the upper part of the gland with the production of virulence proteins and toxins leading to impairment of the host defense power and inflammation of the mammary gland (Foster and Höök, 1998). Of note, most available antibiotics are inefficient in eradicating these infections. To avoid treatment failure in *S. aureus* mastitis, new and alternative therapies in combination with available antimicrobial drugs must be formulated. In recent years, there has been growing interest in the utilization of naturally occurring substances such as essential oils (EOs) manufactured from different plant components owing to their biological effects including antioxidant, anti-inflammatory and anticancer (Krifa et al., 2015). Additionally, EOs have been widely reported in the scientific literature as potential antibacterial agents as they are efficient against a wide variety of pathogenic bacteria and yeast (Swamy et al., 2016; Puškárová et al., 2017). The leaves and inflorescence of *Origanum vulgare* are the main sources for carvacrol essential oil (EO). This plant was used in the ancient alternative medicine as antimicrobial, antidiabetic, anticancer and anti-inflammatory agent (Leyva-Lopez et al., 2017). The monoterpenic phenol such as carvacrol [2-methyl-5-(1-methylethyl) phenol] has significant effects on microbial cell membrane, respiratory metabolism and DNA (Cui et al., 2019). Additionally, it has antivirulence effects on foodborne pathogens, especially *S. aureus* (Cui et al., 2019). Interestingly, linalool, which has antimicrobial and antifungal properties is the main constituent of *Lavandula officinalis* and *Citrus sinensis* EOs (Kasper et al., 2010; Wang et al., 2023). In the same context, eugenol has potential activities against resistant pathogens including bacteria, fungi as well as viral infections. It is found in abundant amounts in clove buds (*Syzygium aromaticum*), cinnamon bark and leaves (*Cinnamomum verum*) (Taleuzzaman et al., 2021).

Relating to the abovementioned issues, the purpose of the current study was to investigate the antimicrobial resistance and virulence profiles of MRSA strains causing mastitis and to evaluate the antimicrobial and antibiofilm activities of different natural compounds including carvacrol, linalool, and eugenol against MRSA strains.

## Materials and methods

### Ethics consideration

All animal care study procedures were conducted in accordance with the guidelines established by the Animal Ethics Review Committee of Suez Canal University (AERC-SCU2023029), Egypt.

### Sampling procedures

The current study enrolled 180 milk samples collected under sterile conditions from 180 different cows suffering from clinical mastitis prior to the beginning of antibiotic treatment from various farms in Sharkia and Ismailia Governorates, Egypt; each sample represented one animal. The udder of each animal was palpated before sample collection to check for edema, heat, asymmetry and other abnormalities. Afterwards, the udder and teats were washed and dried and then 70% ethyl alcohol was used to sanitize the udder, teats and tester hands to remove any chance of contamination. When collecting milk samples, the first few strips were discarded to avoid potential contamination from the teat orifice.

### Microbiological analysis and characterization of *S. aureus* isolates

Firstly, *S. aureus* isolates were isolated onto mannitol salt agar and Baird Parker agar supplemented with an egg yolk–tellurite emulsion (Oxoid, UK). Standard bacteriological procedures were applied to make a preliminary phenotypic identification of *S. aureus* based on their growth on selective media, β-hemolysis on blood agar and production of golden yellow pigments. Furthermore, microscopical examination of Gram stained films from colonies grown onto mannitol salt agar were observed for the formation of Gram-positive grape-like clusters (Becker et al., 2015; Abd El-Hamid et al., 2019). The recovered isolates were then confirmed to be *S. aureus* based on their positive reactions for catalase and coagulase tests. The isolates were finally identified using PCR assay to detect *nuc* gene (Brakstad et al., 1992). All the isolates were preserved frozen in brain heart infusion broth (Oxoid, UK) containing 30% glycerol at - 80°C prior to subsequent detailed analysis.

### Detection of biofilm producing *S. aureus* isolates

*In vitro* formation of biofilms was phenotypically assessed using two methods; qualitative Congo red agar, CRA (Freeman et al., 1989) and quantitative microtiter plate, MTP (Stepanovic et al., 2000) and genotypically via detection of *icaA* gene (Ciftci et al., 2009).

### Antimicrobial susceptibility testing

*In vitro* phenotypic antimicrobial susceptibility profiles of all confirmed *S. aureus* isolates to nine antimicrobial drugs from different groups were assessed on Muller-Hinton agar (Oxoid, UK) using Kirby-Bauer disc diffusion method (Bauer et al., 1966). Standard antibiotic discs (Oxoid, UK) included cefoxitin (CFX), ampicillin (AMP), amoxicillin-clavulanic acid (AMC), erythromycin (E), chloramphenicol (C), sulfamethoxazole-trimethoprim (SXT), ciprofloxacin (CIP), vancomycin (VA) and gentamycin (CN). Clinical and Laboratory Standards Institute (CLSI) interpretation criteria (CLSI, 2020) were used to classify the isolates as either susceptible or resistant depending on the diameter of the inhibition zones surrounding each disc. Using broth microdilution method outlined by CLSI (CLSI, 2020), minimum inhibitory concentrations (MICs) were determined for cefoxitin and vancomycin (Sigma-Aldrich, USA) against all isolates to phenotypically detect MRSA and VRSA, respectively. All isolates showing phenotypic resistance to cefoxitin and vancomycin were subjected to PCR assays for the detection of *mecA* and *vanA* genes specific for MRSA and VRSA, respectively as described elsewhere (Kariyama et al., 2000; Larsen et al., 2008). Multidrug resistant isolates were defined as those exhibiting resistance to at least one agent in three or more different classes of antimicrobial agents.

### Molecular characterization of virulence genes and *agr* genotyping

The presence of *sea, seb, sec, see, hla* and *hlb* virulence genes in addition to *agr* types (I–IV) were determined using singleplex and multiplex PCR assays using EmeraldAmp^®^ GT PCR Master Mix (Takara, USA) and specific primers. The primers used for PCR are listed in **Table 1**. Amplification of target genes was performed as previously stated (Mehrotra et al., 2000; Gilot et al., 2002 and Fei et al., 2011). Controls for each PCR run contained positive (DNA extracted from *S. aureus* reference strain ATCC25923), negative (DNA extracted from *Escherichia coli* reference strain ATCC25922) and no template (PCR reaction mixture components without DNA) samples. Electrophoresis of amplified PCR products on a 1.5% agarose gel stained with ethidium bromide (Sigma-Aldrich, USA) allowed for their visualization under ultraviolet light.

**Table 1:** List of PCR primers and amplicon sizes of target genes investigated in this study.

Target gene	Specificity	Primer sequence (5’-3’)	Amplicon size (bp)	Reference
***nuc***	*S. aureus* species specific	F: GCGATTGATGGTGATACGGTT R: AGCCAAGCCTTGACGAACTAAAGC	270	Brakstad et al., 1992
***mecA***	Penicillin-binding protein	F: TCCAGATTACAACTTCACCAGG R: CCACTTCATATCTTGTAACG	162	Larsen et al., 2008
***vanA***	Vancomycin resistance	F: GTGACAAACCGGAGGCGAGGA R: CCGCCATCCTCCTGCAAAAAA	103	Kariyama et al., 2000
***sea***	Staphylococcal enterotoxin a	F: GGTTATCAATGTGCGGGTGG R: CGGCACTTTTTTCTCTTCGG	102	Mehrotra et al., 2000
***seb***	Staphylococcal enterotoxin b	F: CCAAATAGTGACGAGTTAGG R: AGATGAAGTAGTTGATGTGTATGG	164	
***sec***	Staphylococcal enterotoxin c	F: CACACTTTTAGAATCAACCG R: CCAATAATAGGAGAAAATAAAAG	451	
***see***	Staphylococcal enterotoxin e	F: CTTTTTTTTCTTCGGTCAATC R: GCAGGTGTTGATTTAGCATT	209	
***hla***	Alpha-hemolysin	F: GAAGTCTGGTGAAAACCCTGA R: TGAATCCTGTCGCTAATGCC	704	Fei et al., 2011
***hlb***	Beta-hemolysin	F: CAATAGTGCCAAAGCCGAAT R: TCCAGCACCACAACGAGAAT	496	
***icaA***	Intercellular adhesion protein A	F: CCTAACTAACGAAAGGTAG R: AAGATATAGCGATAAGTGC	315	Ciftci et al., 2009
***agrI***	Accessory gene regulator I	Pan: ATGCACATGGTGCACATGC *agr*I: GTCACAAGTACTATAAGCTGCGAT	441	Gilot et al., 2002
***agrII***	Accessory gene regulator II	pan: ATGCACATGGTGCACATGC *agr*II: TATTACTAATTGAAAAGTGGCCATAGC	575	
***agrIII***	Accessory gene regulator III	pan: ATGCACATGGTGCACATGC *agr*III: GTAATGTAATAGCTTGTATAATAATACCCAG	323	
***agrIV***	Accessory gene regulator IV	Pan: ATGCACATGGTGCACATGC *agr*IV: CGATAATGCCGTAATACCCG	659	

### Essential oils antibacterial and antibiofilm activities

Carvacrol (98% purity), linalool (97% purity) and eugenol (99% purity) EOs purchased from Sigma-Aldrich Corporation (St. Louis, MO, USA) were evaluated for their antibacterial activities against MDR and multivirulent MRSA and VRSA isolates using an agar well diffusion assay (Elmowalid et al., 2022). The stock solutions of EOs were prepared in 10% dimethyl sulfoxide as a diluent, since it is a known universal solvent with no antibacterial activity at this concentration; it was used as a negative control in one well of each tested plate. Positive results were recorded as zones of inhibition of > 7 mm. Subsequently, MIC values of the screened EOs were evaluated via broth microdilution method (Elmowalid et al., 2022). Notably, the effects of investigated EOs at their sub-MIC (0.5 MIC) levels on the biofilms of the tested isolates were further assessed via CRA (Freeman et al., 1989) and MTP (Stepanovic et al., 2000) methods. Two sets were performed, in triplicate, for each isolate in control plates with plain media and in plates with media and sub-MIC levels of the EOs.

### Interaction of essential oils with antimicrobials via checkerboard assay

Evaluating the *in vitro* interaction between EOs and the least effective antimicrobials against MDR and multivirulent MRSA and VRSA isolates was done using a checkerboard technique, in triplicate, adopting the protocol previously detailed (Magi et al., 2015). Assessing the interaction between the investigated antimicrobial compounds was conducted through calculating the fractional inhibitory concentration index (FICI) using the following formula: FICI = MIC of the least effective antimicrobial in combination/MIC of antimicrobial alone + MIC of EO in combination/MIC of EO alone. The obtained FICI values were interpreted as following: synergism; FICI ≤ 0.5, additivity; 0.5 < FICI ≤ 1, indifference; 1 < FICI ≤ 4 and antagonism; FICI > 4.

### Molecular docking studies

The molecular docking program MOE 2019 suite was used to investigate the antibiofilm potential of the three EOs against *S. aureus* (Inc, 2016). The molecular docking was established for Bap of *S. aureus*. The PerkinElmer ChemOffice Suite 2017 was used to determine and draw the chemical structures of the assessed compounds, which were then available for the molecular docking process (Elmaaty et al., 2021). These compounds were introduced into one database to be downloaded as an MDB extension file. Moreover, the X-ray structure of *S. aureus* Bap was downloaded from the online RCSB website with PDB entry: 7c7u (Ma et al., 2021). Accordingly, selected protein was prepared for molecular docking as previously discussed (Ma et al., 2021).

### Statistical analysis

Significant variations were detected using Chi- square without replication. Typical statistically significant results were identified when the *p* value was < 0.05. All dendrograms and figures were constructed using the R packages corrplot, heatmap, hmisc, and ggpubr (Galili et al., 2018).

## Results

### Characterization of biofilm producing *S. aureus* isolates

Phenotypic analysis of mastitis milk samples revealed 37 staphylococcal isolates (20.6%), which were all confirmed to be *S. aureus* based on standard conventional bacteriological tests in addition to genetic detection of *nuc* (*S. aureus* species-specific) gene. Of note, all recovered isolates were identified phenotypically as biofilm producers depending on growth onto Congo red agar and adherence on MTP and genotypically via their possession for *ica*A gene.

### Antimicrobial susceptibility results

**Figure 1:**
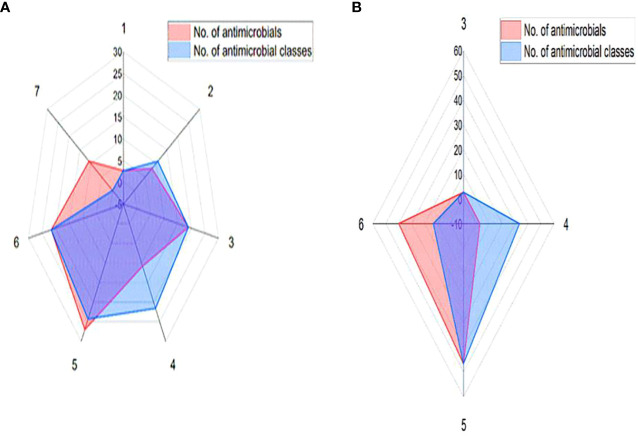
Antimicrobial resistance patterns of MRSA **(A)** and VRSA **(B)** isolates.

The recovered 37 *S. aureus* isolates exhibited full resistance to cefoxitin (100%) and high resistance rates were detected against ampicillin (81.1%), followed by erythromycin (67.6%) and gentamycin (62.2%). Meanwhile, higher sensitivity rate was detected against ciprofloxacin (78.4%). There were statistically significant (*p* < 0.05) variations in the susceptibility patterns among *S. aureus* isolates against various antimicrobials. All 37 phenotypically cefoxitin resistant *S. aureus* isolates were positive for *mec*A gene; thus, molecularly confirmed as MRSA. Basing on phenotypic vancomycin resistance, 15 out of 37 (40.5%) *S. aureus* isolates were positive for *van*A gene being defined as VRSA. Of note, 73% (27/37) of the tested MRSA isolates and all VRSA ones were MDR (**Figure 1**).

### Molecular investigation of virulence genes and *agr* genotyping

**Figure 2:**
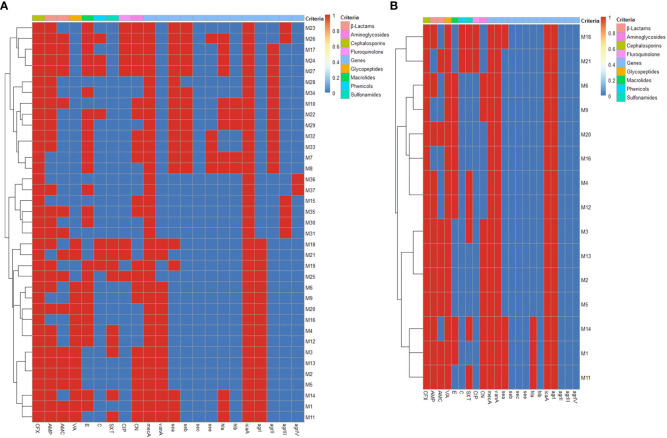
Heat maps illustrating the distribution of phenotypic antimicrobial resistance, *mecA*, *vanA* and virulence genes and *agr* genotypes among MRSA **(A)** and VRSA **(B)** isolates. The blue and red colors represent the sensitivity and resistance to certain antimicrobial agent and the absence and presence of *mecA*, *vanA* and certain virulence gene and *agr* genotype, respectively. The recovered isolates are coded on the right of the heat map; M: mastitis milk. CFX: cefoxitin, AMP: ampicillin, AMC: amoxicillin-clavulanic acid, VA: vancomycin, E: erythromycin, C: chloramphenicol, SXT: sulfamethoxazole-trimethoprim, CIP: ciprofloxacin and CN: gentamycin, *mecA*: methicillin resistance encoding gene, *vanA*: vancomycin resistance encoding gene, *sea*: staphylococcal enterotoxin a gene, *seb*: staphylococcal enterotoxin b gene, *sec*: staphylococcal enterotoxin c gene, *see*: staphylococcal enterotoxin e gene, *hla*: alpha-hemolysin gene, *hlb*: beta-hemolysin gene, *icaA*: intercellular adhesion A gene, *agr*: accessory gene regulator gene.

All our isolates were positive for *ica*A gene (100%), while no isolates were positive for *sec* gene. Furthermore, *sea, seb*, *see*, *hla* and *hlb* genes were more prevalent among MRSA isolates (51.3, 35.1, 13.5, 32.4 and 13.5%) than VRSA ones (26.7, 0, 0, 20 and 0%), respectively (**Figure 2**). Besides, 16 MRSA (43.2%) and 3 VRSA (20%) isolates were multivirulent harboring three or more virulence genes (**Figure 2**). There were statistically significant (*p* < 0.05) variations in the occurrence of virulence genes among MRSA and VRSA isolates. Concerning *agr* genotyping, majority of MRSA (45.9%) and all VRSA (100%) isolates were positive for *agrI* gene. Furthermore, *agrII*, *agrIII* and *agrIV* genes were more prevalent among MRSA (32.4, 16.2 and 5.4%).

### *In vitro* antibacterial and antibiofilm activities of the tested essential oils

The antibacterial potentials of carvacrol, linalool, and eugenol EOs were investigated against MDR and multivirulent MRSA strains. Considering the zones of inhibition and MIC values, these natural compounds exhibited excellent antibacterial efficacy against all investigated isolates with relevant inhibition zones’ diameters ranging from 20 to 37 mm and MIC values of 0.5 - 8 µg/mL. In contrast to eugenol, linalool EO showed the highest antimicrobial activities with mean inhibition zones’ diameters of 24 ± 0.5 mm and MIC values between 0.5 and 2 µg/mL.

Regarding the antibiofilm activities of carvacrol, linalool, and eugenol EOs, pronounced effects were noticed against the examined isolates. Linalool EO showed the highest antibiofilm activities, followed by carvacrol then eugenol (*p* < 0.5). This was evidenced by prominent reduction in the capacity of all tested biofilm producing isolates post exposure to the screened EOs comparing with the untreated ones with inhibitory capacity percentages fluctuating from 98.90 to 99.96%.

### Assessing interaction between antimicrobials and essential oils

Owing to the full resistance of tested MRSA and VRSA isolates to cefoxitin and vancomycin, respectively, the activities of both antibiotics were examined in combination with the screened EOs. The results of checkerboard assay exhibited noteworthy synergistic interactions between these antibiotics and the investigated EOs against all MRSA and VRSA isolates with FIC values ≤ 0.5. Fortunately, the antimicrobial resistance patterns of 13 isolates; 10 MRSA and 3 VRSA changed to cefoxitin and vancomycin sensitive phenotypes upon treating with any of the investigating EOs, respectively.

### Molecular docking results

**Figure 3:**
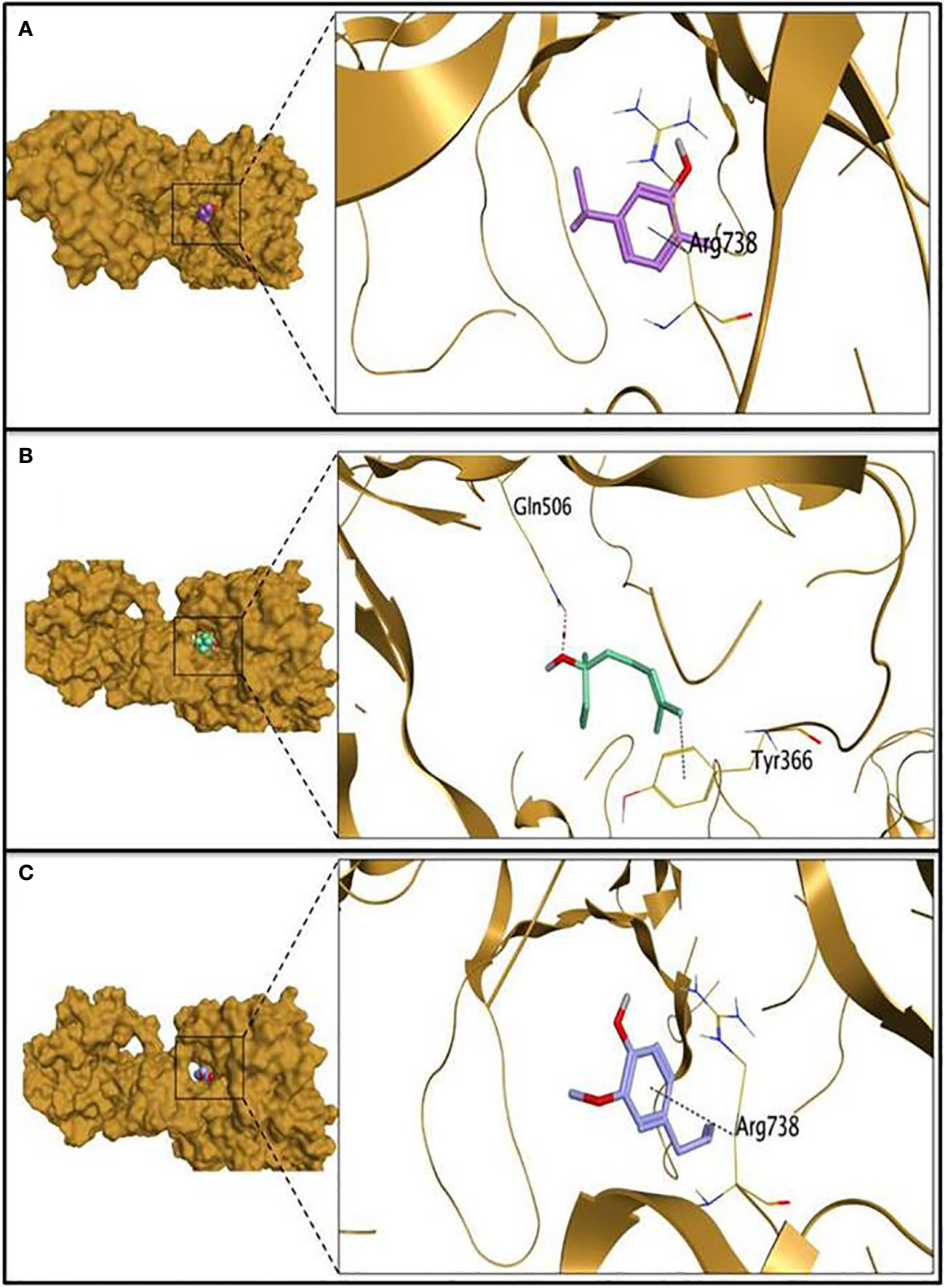
The 3D protein positioning and 3D binding interactions of the tested essential oils with *S. aureus* biofilm associated protein; binding of carvacrol **(A)**, linalool **(B)** and eugenol **(C)** with PDB entry of 7c7u.

The molecular docking study was carried out to evaluate the potential of the three tested EOs against *S. aureus* biofilms getting far deep understanding and further insights about their antibiofilm activities. According to molecular docking results, linalool showed the highest binding capacity, followed by carvacrol then eugenol. Linalool could make a stable complex with a binding energy of -6.00 Kcal/mol at root mean square deviation (RMSD) value of 1.32 Å. It was disclosed that the hydroxyl group of linalool could form H bond with GLN506 at a distance of 3.15 Å. Additionally, the terminal methyl group at position 8 of linalool could form H-pi with TYR366 at a distance of 3.78 Å as depicted in **Table 2** and **Figure 3**. Besides, it was shown that carvacrol could make a stable complex with Bap with a binding energy of -5.59 Kcal/mol at an RMSD value of 1.09 Å. It was found that the phenyl moiety of carvacrol could form pi-H bond with ARG738 at a distance of 3.73 Å (**Table 2** and **Figure 3**). Moreover, eugenol was able to form a stable complex with a binding energy of -5.44 Kcal/mol at an RMSD value of 0.64 Å. It was revealed that the phenyl ring of eugenol could form pi-H bond with ARG738 at a distance of 4.62 Å as represented in **Table 2** and **Figure 3**.

**Table 2:** Ligand-protein complex binding energy, RMSD and binding interactions of carvacrol, linalool and eugenol EOs with *S. aureus* biofilm associated protein.

EO	S Score (Kcal/mol)	RMSD (Å)	Binding interaction	Distance (Å)
**Carvacrol**	-5.59	1.09	ARG738/pi-H	3.73
**Linalool**	-6.00	1.32	GLN506/H-acceptor; TYR366/H-pi	3.15 3.78
**Eugenol**	-5.44	0.64	ARG738/pi-H	4.62

RMSD, root mean square deviation.

## Discussion

Bovine mastitis as one of the most important dairy cattle diseases affecting mammary tissue may be chronic, clinical or subclinical leading to great economic losses. Of note, serious zoonotic diseases were always associated with different form of mastitis (Hoe and Ruegg, 2005). Moreover, mastitis shares in the wide spread of antimicrobial resistance globally (Beyene et al., 2017). The resistant biofilm producing *S. aureus* is the most common cause of bovine mastitis; therefore, new therapeutic options including complementary and alternative therapies are urgently required (Ghaly et al., 2021; Mosallam et al., 2021; Elfaky et al., 2022; Ghaly et al., 2023). So, we aimed to find successful antimicrobial protocols to prevent the wide spreading of mastitis that is particularly associated with biofilm producing *S. aureus* in the endemic area.

In this study, we recorded relative higher prevalence of bovine mastitis with *S. aureus* infections (20.6%). Other studies announced lower prevalence rates of *S. aureus* (16.1, 10 and 3%) among mastitic cows (Schukken et al., 2009; Tenhagen et al., 2009; Beyene et al., 2017). The variations in the prevalence rates of *S. aureus* among mastitic cows in this study and other previous studies may be attributed to differences in the standard hygienic practices applied in different countries (Getaneh and Gebremedhin, 2017). Therefore, proper control practices should be directed to prevent the wide spreading of bovine mastitis through segregations or selective culling of infected animals in some cases, which did not respond to any type of treatment protocols side by side with proper milking procedures (Ruegg, 2017). Interestingly, poor prognosis is always associated with the bacterial pathogens with multivirulence arrays (Ammar et al., 2020). Biofilm production is one of the main causes of antimicrobial resistance and it is a leading trait for increasing the sharpness and frequency of bovine mastitis treatment failure. Biofilm producing *S. aureus* is the causative agent of severe mastitis cases that respond very slowly to treatment (Szweda et al., 2012; Abd-El-Hamid et al., 2020). In this study, all our *S. aureus* isolates were phenotypically and genotypically identified as biofilm producers. The phenotypic identification of biofilm producers matched with the genetic detection of *icaA* gene among all *S. aureus* isolates. Previously, almost all *S. aureus* isolates causing bovine mastitis were able to produce biofilms (de Castro Melo et al., 2013; Bendary et al., 2016; Notcovich et al., 2018).

Of note, all our isolates were identified as MRSA, which showed complete resistance to cefoxitin and harbored *mecA* gene. Unfortunately, VRSA were detected among our isolates with a relative higher prevalence rate (40.5%). Convergently, previous reports announced that MRSA were the most prevalent recovered strains among bovine mastitis (Holmes and Zadoks, 2011; Krukowski et al., 2020). Moreover, 73% (27/37) of the tested MRSA isolates and all VRSA ones were MDR. Of note, the antimicrobial resistance and the wide spreading of MDR isolates were increasingly noticeable among bovine mastitis cases worldwide (Hoque et al., 2018; Salauddin et al., 2020). The antimicrobial resistance is a global multifaceted phenomenon and the increasing rates of this problem may be attributed to several factors including the inappropriate use of antibiotics, especially in veterinary fields as growth enhancers, self-medication and the poor application of antimicrobial stewardship programs (Prestinaci et al., 2015).

Surprisingly, most of our isolates were multivirulent harboring three or more virulence genes, which may compound the severity of the diseases. Therefore, bovine mastitis associated with biofilm producing MRSA showing MDR patterns is a common crisis globally. Therefore, there is an urgent need for additional efforts and researches to address this issue. Poor prognosis is always associated with biofilm producing MRSA strains owing to the extreme resistance of microbial cells in biofilms. For that, finding new alternatives are important research approaches, which have attracted the interest of many researchers. One of innovative approaches to treat *S. aureus* biofilm-related infections was evaluated in previous studies using non-antibiotics drugs (Kiedrowski and Horswill, 2011; Richter et al., 2017). Some detergent such as cis-2-decanoic acid could disperse staphylococcal biofilms (Davies and Marques, 2009). Moreover, EOs such as carvacrol, linalool and eugenol have been used in food industry owing to their preservative potency against foodborne pathogens. In this report, we evaluated the antimicrobial and antibiofilm activities of these EOs against *S. aureus*, the predominant contagious pathogens causing bovine mastitis and we found great anti-MRSA activities of the investigated EOs, especially linalool. This finding goes parallel with the published results for linalool antimicrobial and antibiofilm activities (Bagamboula et al., 2004; Aelenei et al., 2019). The cell membrane, especially mesosomes and cell wall integrity are the targeting sites of linalool (Gao et al., 2019). Interestingly, the synergistic interactions between the investigated EOs and other antimicrobial drugs were announced in this study. Several authors stated that treatment failure owing to antimicrobial resistance could be solved via using combinations of the available antibiotics and other natural compound such as EOs (Lahmar et al., 2017; Aelenei et al., 2019; Özel et al., 2022). These EOs could increase the rates of antimicrobial susceptibility and reversal of antimicrobial resistance. The antibiotic actions could be rescued when used in combination with EOs. In accordance with our results, the resistances of MRSA to β-lactam antibiotics were highly reduced in the presence of EOs (Lahmar et al., 2017). Although the exact synergistic interactions between antibiotics and EOs have still not been exactly clarified, several authors used time-kill curve analysis to confirm the efficacy of these combinations (Aleksic et al., 2014; Abd El-Hamid et al., 2022).

## Conclusion

Multidrug resistance and multivirulence were the common phenotypes among MRSA strains incriminated in bovine mastitis. Despite the great difficulties to control and eradicate these phenotypes with common used antimicrobial drugs, EOs, especially linalool as proven in this study give us the bright hope to increase the therapeutic options and the possibility of treatment success. Therefore, we recommended using combination therapies between the available antibiotics and the natural compounds such as EOs.

## Data availability statement

The original contributions presented in the study are included in the article/supplementary material. Further inquiries can be directed to the corresponding author.

## Ethics statement

Collection of milk samples in this study was approved by the Animal Ethics Review Committee of Suez Canal University (AERCSCU2023029), Egypt.

## Author contributions

MA: Methodology, Software, Writing – review & editing. RE: Investigation, Formal Analysis, Writing – review & editing. MBa: Data curation, Project administration, Writing – review & editing. MA: Software, Visualization, Writing – review & editing. AS: Data curation, Formal Analysis, Writing – review & editing. KA: Funding acquisition, Data curation, Writing – review & editing. FS: Supervision, Writing – review & editing. AM: Supervision, Formal Analysis, Writing – review & editing. NA: Conceptualization, Validation, Writing – review & editing. MG: Resources, Supervision, Writing – review & editing. AE: Methodology, Software, Writing – review & editing. HA: Formal Analysis, Writing – review & editing. MBe: Methodology, Writing – original draft, Writing – review & editing.
